# Simultaneous enrichment and sequential separation of glycopeptides and phosphopeptides with poly-histidine functionalized microspheres

**DOI:** 10.3389/fbioe.2022.1011851

**Published:** 2022-10-06

**Authors:** Danyi Shang, Cheng Chen, Xuefang Dong, Yun Cui, Zichun Qiao, Xiuling Li, Xinmiao Liang

**Affiliations:** ^1^ Key Laboratory of Separation Science for Analytical Chemistry, Dalian Institute of Chemical Physics, Dalian, China; ^2^ University of Chinese Academy of Sciences, Beijing, China; ^3^ Ganjiang Chinese Medicine Innovation Center, Nanchang, China

**Keywords:** poly-histidine, simultaneous enrichment, sequential elution, phosphopeptides, glycopeptides

## Abstract

Protein phosphorylation and glycosylation coordinately regulate numerous complex biological processes. However, the main methods to simultaneously enrich them are based on the coordination interactions or Lewis acid-base interactions, which suffer from low coverage of target molecules due to strong intermolecular interactions. Here, we constructed a poly-histidine modified silica (SiO_2_@Poly-His) microspheres-based method for the simultaneous enrichment, sequential elution and analysis of phosphopeptides and glycopeptides. The SiO_2_@Poly-His microspheres driven by hydrophilic interactions and multiple hydrogen bonding interactions exhibited high selectivity and coverage for simultaneous enrichment of phosphopeptides and glycopeptides from 1,000 molar folds of bovine serum albumin interference. Furthermore, “on-line deglycosylation” strategy allows sequential elution of phosphopeptides and glycopeptides, protecting phosphopeptides from hydrolysis during deglycosylation and improving the coverage of phosphopeptides. The application of our established method to HT29 cell lysates resulted in a total of 1,601 identified glycopeptides and 694 identified phosphopeptides, which were 1.2-fold and 1.5-fold higher than those obtained from the co-elution strategy, respectively. The SiO_2_@Poly-His based simultaneous enrichment and sequential separation strategy might have great potential in co-analysis of PTMs-proteomics of biological and clinic samples.

## 1 Introduction

Protein post-translational modifications (PTMs) refer to covalent addition of functional groups to specific amino acid residues in a protein (Macek et al., 2019). There are 469 types of PTMs have been reported (Prabakaran et al., 2012). These PTMs not only affect protein three-dimensional structures but also jointly regulate numerous biological processes *in vivo* (Aebersold et al., 2018; Harmel and Fiedler, 2018). It is reported that multiple PTMs occurring on a protein often work interdependently, which is termed as “crosstalk” (Venne et al., 2014; Cuijpers and Vertegaal, 2018). Protein phosphorylation and glycosylation, two of the most ubiquitous and important PTMs, undergo crosstalk and play crucial roles in numerous cellular processes (Vu et al., 2018). Deregulation of crosstalk between phosphorylation and glycosylation has been implicated in many severe human diseases, such as autoimmune disease, Alzheimer’s disease and cancers (Jennewein and Alter, 2017; Aasen et al., 2018; Wu et al., 2019). The PTMs crosstalk is regulated in an antagonistic or synergetic manner. For example, the glycosylation of epidermal growth factor receptor (EGFR) inhibits the abnormal phosphorylation of EGFR in TKI-resistant lung cancer cell line (Yen et al., 2015); the N-glycosylation at N359 ameliorated the hyperphosphorylated and aggregated of tau (Losev et al., 2021). Shedding light on PTMs crosstalk will help us better understand etiology and provide novel targets for drug therapy. Therefore, it is necessary to simultaneously study the protein glycosylation and phosphorylation and reveal the crosstalk between them at the molecular level.

Mass spectrometry (MS) is a mainstreamed analysis method in proteomics (Calderon-Celis et al., 2018; Xiao et al., 2018). However, direct analysis of protein PTMs crosstalk on the proteome level is quite challenging owing to the low abundance of PTM peptides, as well as significant background interference deriving from complex mixture in the biosamples. Besides, some clinic or biological samples are too precious to suffer from more than one pretreatment and co-analyze different types of protein PTMs is required to obtain as much information as possible in one sample pretreatment. Hence, it is urgently needed to develop strategies to simultaneously and selectively enrich PTM peptides prior to MS analysis.

In recent years, several methods have been developed to simultaneously enrich glycopeptides and phosphopeptides, which mainly include metal oxide affinity chromatography (MOAC, represented by TiO_2_) (Luo et al., 2019; Chu et al., 2022; He et al., 2022), immobilized metal ion affinity chromatography (IMAC, typical example is Ti^4+^-IMAC) (Wang et al., 2019; Zhang et al., 2020; Zheng et al., 2020; Huang et al., 2021) and hydrophilic interaction liquid chromatography (HILIC) (Lu et al., 2020; Luo et al., 2021). For MOAC, TiO_2_ was firstly used to simultaneously enrich phosphopeptides and glycopeptides in our group, based on the Lewis acid-base interaction between the PTM peptides and the adsorbents (Yan et al., 2010). However, the interaction between PTM peptides and TiO_2_ is too strong to elute multi-phosphopeptides or multiply sialylated glycopeptides (Lu et al., 2020). In order to improve the enrichment coverage of the absorbent, other hydrophilic functional groups have been introduced to the TiO_2_ surface (Sun et al., 2019; Sun et al., 2020; Yi et al., 2020). IMAC utilizes the chelation interaction between the immobilized metal cations and the PTM peptides. However, the unmodified peptides with multiple acidic amino acids could be captured by IMAC materials, interfering the PTM peptides enrichment. To improve enrichment selectivity, hydrophilic groups are combined with metal cations in the functional materials for simultaneously enriching glycopeptides and phosphopeptides, such as hydrophilic chitosan and phytic acid. (Zou et al., 2017; Hong et al., 2018). Both MOAC- and IMAC-based simultaneous enrichment materials utilize the strong interaction between materials and PTM peptides. While HILIC materials based on the multiple hydrogen bonding interactions provide an alternative for simultaneous enrichment of phosphopeptides and glycopeptides (Lu et al., 2020; Luo et al., 2021). Benefited from the multiple hydrogen bonding interactions between adsorbent and target peptides, the HILIC-based simultaneous enrichment materials have high selectivity and recovery. Encouraged by these studies, it is worthwhile to develop more tunable materials based on multi-hydrogen bonding for the simultaneous enrichment of phosphopeptides and glycopeptides.

Concerning to the routine protocol for glycosylation site analysis, the enriched N-linked glycopeptides need to be deglycosylated with PNGase F in alkaline aqueous solution before MS analysis. But phosphopeptides may be hydrolyzed under this pH condition (Li et al., 2014), which could lead to loss of some phosphorylation information. Some researchers utilized the method of sequential PTM peptides enrichment to simultaneously enrich glycopeptides and phosphopeptides (Cho et al., 2019; Andaluz Aguilar et al., 2020; Zhou et al., 2020). First, one type of PTM peptides was enriched by the first enrichment strategy from peptide mixtures, and then flow-through from the first enrichment is enriched for another type of PTM peptides using another enrichment method. Although the sequential PTM peptides enrichment methods can separate glycopeptides and phosphopeptides and avoid this problem, the large amounts of starting samples and the cumbersome operation process are required. Therefore, it will be ideal that simultaneous enrichment and sequential separation of phosphopeptides and glycopeptides can be performed with the same materials.

Histidine (His) is a naturally amphiphilic amino acid, which may interact glycopeptides through multiple hydrogen bonding and electrostatic interactions (Dong et al., 2017). Unlike other amino acids, His has a unique imidazole side chain structure, which interact glycan through CH-π interactions (Kiessling and Diehl, 2021). Moreover, the hydrogen bonding interactions between His and phosphate groups have been reported between DNA and His (Chattopadhyay et al., 2016), between triosephosphate isomerase and His (Lodi and Knowles, 1991), and between nucleic acids and His (Chou et al., 2014). Encouraged by these reports, we prepared poly-histidine modified silica (SiO_2_@Poly-His) microspheres for efficiently co-enriching glycopeptides and phosphopeptides. Meanwhile, we developed an on-line deglycosylation strategy for sequential elution of glycopeptides and phosphopeptides. Firstly, the glycopeptides and phosphopeptides were simultaneously captured by SiO_2_@Poly-His microspheres; then the glycopeptides that absorbed on microspheres were on-line deglycosylated with PNGase F; the deglycosylated peptides and phosphopeptides were sequentially eluted, and analyzed by MS respectively. The enrichment performance of the SiO_2_@Poly-His microspheres to glycopeptides and phosphopeptides were systematically evaluated, and the optimal conditions for the entire protocol were optimized. Finally, the SiO_2_@Poly-His microspheres were used to simultaneously enrich phosphopeptides and glycopeptides from the tryptic digests of standard proteins and from a complex HT29 cell lysate.

## 2 Materials and methods

### 2.1 Reagents

Acrylated histidine (AA-His, >95%) was obtained from China-Peptides Corp. (Shanghai, China). Acetonitrile (ACN, HPLC-grade), formic acid (FA, 98%), acetic acid (AA, 99.7%), urea (>99.0%), 2,2′-azobis (2-methylpropionamide) dihydrochloride (AIBA, 97%), 4-cyano-4-(phenylcarbonothioylthio)pentanoic acid (CPADB, 97%), ammonium hydroxide (NH_3_·H_2_O, 28%–30% NH_3_), ammonium bicarbonate (NH_4_HCO_3_, 99.0%), iodoacetamide (IAA, 99.0%), DL-dithiothreitol (DTT, 99.0%), ammonium acetate (CH_3_COONH_4_, 99.0%), glycolic acid (99.0%), [Glu1]-Fibrinopeptide B human (GFB), bovine serum albumin (BSA, >98%), α-casein (>98%), bovine fetuin (>99.9%) and trypsin (>98%) were purchased from Sigma Aldrich (St Louis, United States). N-hydroxysuccinimide (NHS; 98%) and dicyclohexylcarbodiimide (DCC; 99%) were bought from Shanghai Macklin Biochemical Co., Ltd. (Shanghai, China). Acetone (≥99.7%), and ethyl alcohol (≥99.7%) was purchased from Sinopharm Chemical Reagent Co., Ltd. (Beijing, China). Standard phosphopeptide (with sequence of HS*PIAPSSPSPK) was obtained from Qiangyao Biotechnology Co., Ltd. (Shanghai, China). PNGase F was purchased from New England Biolabs (Ipswich, United States). Radioimmunoprecipitation (RIPA) lysis buffer was purchased from Beyotime Biotechnology (Shanghai, China). Bicinchoninic acid (BCA) protein assay kit was purchased from Thermo Fisher scientific (CA, United States). TiO_2_ was bought from GL Sciences (Tokyo, Japan). C18HC material and amino silica (5 μm, 300 Å) materials were purchased from ACCHROM (Wenling, China). Water was purified by a Milli-Q system (Millipore, Milford, United States).

### 2.2 Instruments

Scanning electron microscopy (SEM) image was taken on a JEM-7800F (JEOL Company, Japan) instrument. Zeta potential was measured by Malvern Zetasizer Nano ZS (Malvern, United Kingdom) at 25°C. N_2_ adsorption–desorption measurement was obtained using QUADRASORB SI (QuantaChrome, United States). Thermogravimetric analysis was performed using a STA449F5 thermostar (NETZSCH, Germany). Protein/peptide concentration was determined by a Multiskan™ FC microplate reader (Thermo Scientific, United States). Infrared spectroscopy was measured by HYPERION 3000 (Bruker Optics, Germany). Standard protein digests were qualitatively analyzed using nano-electrospray ionization quadrupole time-of-flight mass spectrometry (nano-ESI-Q-TOF MS) (Waters, United Kingdom). The peptides extracted from the HT29 cell line were qualitatively analyzed using an EASY-nLC 1,200 liquid chromatography system and an Orbitrap Exploris 480 mass spectrometer (Thermo Scientific, United States).

### 2.3 Synthesis of poly histidine modified silica microspheres

The CPADB functionalized silica microspheres (SiO_2_-CPADB) were synthesis according to the reported literature (Khani et al., 2017). AA-His (128 mg, 0.6 mmol), SiO_2_-CPADB with surface density of 0.30 mmol/g (0.1 g, 0.03 mmol), ethanol (2.2 ml), and 10 mM sodium acetate solution (2.2 ml), AIBA initiator (0.03 mmol) with a ratio between species of [monomer]:[CTA]:[initiator] = 20:1:1 were added to a round bottomed flask. The mixture was degassed through three freeze pump-thaw cycles, injected with nitrogen, and then the unit was placed in an oil bath with agitation at 55°C for 72 h. The obtained silica was washed three times with water before being centrifuged at 5,000 rpm for 3 minutes and dried for storage.

### 2.4 Retention of glycopeptides on SiO_2_@Poly-His microspheres

1 mg of SiO_2_@Poly-His or SiO_2_-NH_2_ pellets were packed into GELoader tips and 10 μg of the bovine fetuin digests that redissolved in 40 µl of 80% acetonitrile (ACN)/1% formic acid (FA) was loaded into the microcolumn. The microcolumn was subsequently eluted with 80% ACN/1% FA (40 µl), 70% ACN/1% FA (40 µl), 60% ACN/1% FA (40 µl), 50% ACN/1% FA (40 µl), respectively. The eluates were collected and analyzed by nano-ESI-Q-TOF MS.

### 2.5 Investigation of phosphopeptides retention mechanism

#### 2.5.1 Effect of acetonitrile content on retention of phosphopeptides

1 mg of SiO_2_@Poly-His microspheres were packed into GELoader tips and 2 μg of the α-casein digests that redissolved in 40 µl of 80% ACN/1% FA was loaded into the microcolumn. The microcolumn was subsequently eluted with 80% ACN/1% FA (40 µl), 70% ACN/1% FA (40 µl), 60% ACN/1% FA (40 µl), 50% ACN/1% FA (40 µl), 40% ACN/1% FA (40 µl), respectively. The eluates were collected and analyzed by nano-ESI-Q-TOF MS.

#### 2.5.2 Effect of FA content on retention of phosphopeptides

1 mg of SiO_2_@Poly-His microspheres were packed into GELoader tips and 2 μg of the α-casein digests that redissolved in 40 µl of 70% ACN/0.1% FA was loaded into the microcolumn. The microcolumn was subsequently eluted with 70% ACN/0.1% FA (40 µl), 70% ACN/0.5% FA (40 µl), 70% ACN/1% FA (40 µl), 70% ACN/2% FA (40 µl), 70% ACN/5% FA, respectively. The eluates were collected and analyzed by nano-ESI-Q-TOF MS.

### 2.6 Enrichment of glycopeptides and phosphopeptides from standard proteins

#### 2.6.1 Glycopeptides enrichment

Tryptic digests of bovine fetuin and BSA were dissolved in 200 μl binding buffer (83% ACN/1% FA) and then 1 mg of SiO_2_@Poly-His microspheres were added and incubated at room temperature for 15 min. After centrifugation, the supernatant was removed and the SiO_2_@Poly-His microspheres were washed with 80% ACN/1% FA and 70% ACN/1% FA to remove non-glycopeptides. The glycopeptides that adsorbed on the poly His microspheres were eluted by 40% ACN/5% FA. The eluates were collected and analyzed by nano-ESI-Q-TOF MS.

#### 2.6.2 Phosphopeptides enrichment

Tryptic digests of α-casein and BSA were dissolved in 200 μl binding buffer (83% ACN/1% FA) and then 1 mg of SiO_2_@Poly-His microspheres were added and incubated at room temperature for 15 min. After centrifugation, the supernatant was removed and the SiO_2_@Poly-His microspheres were washed with 80% ACN/1% FA and 75% ACN/1% FA to remove non-phosphopeptides. The phosphopeptides adsorbed on the poly His were eluted by 40% ACN/5% FA. The eluates were collected and analyzed by nano-ESI-Q-TOF MS.

#### 2.6.3 Glycopeptides and phosphopeptides simultaneous enrichment

Tryptic digests of bovine fetuin, α-casein and BSA were dissolved in 200 μl binding buffer (83% ACN/1% FA) and then 1 mg of SiO_2_@Poly-His microspheres were added and incubated at room temperature for 15 min. After centrifugation, the supernatant was removed, and the SiO_2_@Poly-His microspheres were washed with 80% ACN/1% FA and 75% ACN/1% FA to remove unmodified peptides. The glycopeptides and phosphopeptides adsorbed on the poly His were eluted by 40% ACN/5% FA. The eluates were collected and analyzed by nano-ESI-Q-TOF MS.

### 2.7 Hydrolysis degree of phosphopeptides during the deglycosylation process

The standard phosphopeptide was dissolved in 50 mM ammonium bicarbonate solution and 5U PNGase F was added to react at 37°C for 0, 3, 6, 9, and 12 h, respectively.

### 2.8 On-Line deglycosylation of the glycopeptides

The poly His microspheres adsorbed with glycopeptides and phosphopeptides were mixed with 5 μL of PNGase F (2,500 U) in 30 μl of 5 mM CH_3_COONH_4_. The entire suspension mixture was incubated for 1 h at 37°C. The resulting mixture was concentrated and redissolved in 80% ACN/0.1% FA. After packing the entire mixture into the tip column, the flow-through was collected and the SiO_2_@Poly-His microspheres were eluted with 80% ACN/1% FA and 40% ACN/5% FA, respectively. The 80% ACN/1% FA fraction and flow-through fraction were combined, and all fractions were analyzed by nano-ESI-Q-TOF MS.

### 2.9 Simultaneous enrichment of glycopeptides and phosphopeptides from HT29 cell line lysate

To culture HT29 cell line, McCoy’s 5A Medium and 10% fetal bovine serum were added and mixed at 37°C with 5% CO_2_. The protein extraction steps were referred to the previous literature (Lu et al., 2020). HT29 cell line lysate (100 μg) was digested by trypsin and Glu C, desalted and dissolved in 100 μl of 83% ACN/1% FA, and then a poly His microsphere (1 mg) was added and incubated for 30 min at room temperature. After centrifugation, the pellet was washed with 83% ACN/1% FA and 80% ACN/1% FA to remove unmodified peptides. The supernatant was discarded by centrifugation and the pellet was mixed with 5 μl of PNGase F (2,500 U) in 30 μl of 5 mM CH_3_COONH_4_, incubated for 1 h at 37°C. The resulting mixture was concentrated and redissolved in 80% ACN/0.1% FA. After packing the entire mixture into the GELoader tips, the flow-through was collected and the SiO_2_@Poly-His microspheres were eluted with 80% ACN/1% FA and 40% CAN/5% FA, respectively. The 80% ACN/1% FA fraction and sample solution fraction were combined, and all fractions were analyzed by Orbitrap Exploris™ 480 Mass Spectrometer.

### 2.10 MS Analysis

#### 2.10.1 Analysis of glycopeptides and phosphopeptides from standard proteins with MS

The enriched glycopeptides from bovine fetuin and phosphopeptides from α-casein were analyzed by a nano-ESI-Q-TOF MS with collision-induced dissociation in a positive mode. The source temperature was set to 100°C and the capillary voltage was set to 2.1 kV. The full scan range was from 600 to 1800 m/z.

#### 2.10.2 Analysis of glycopeptides and phosphopeptides from HT29 cell line lysate with LC-MS

The enriched PTM-peptides were separated and identified on EASY-nLC 1,200 liquid chromatography system coupled with Orbitrap Exploris™ 480 mass spectrometer. A C18 analytical column (150 μm × 150 mm, 2 μm) was used to separate PTM-peptides. The mobile phase A was 0.1% FA and phase B was 80% ACN/0.1% FA. The gradient elution was as follows: 12–30% B, 62 min; 30%–38% B, 10 min; 38%–95% B, 8 min; and 95% B, 10 min. The flow rate was 600 nL/min. The parameter of Orbitrap Exploris™ 480 mass spectrometer was set as follows; the capillary temperature of the ion transport, 320°C; the spray voltage, 2.1 kV. The MS was operated in positive mode with the FAIMS Pro interface. Compensation voltage was set at −45 V and −65 V to remove singly charged ions. For data-dependent acquisition (DDA) experiments full MS resolution was set at 60,000 with a normalized AGC target 300%. The full scan range was from 350 to 1,500 m/z and a maximum inject time was set at 20 ms. The RF Lens was set at 50%. For MS2, resolution was set at 15,000 with a normalized AGC target of 75%. The maximum inject time was set at 30 ms. The data-dependent MS/MS was top speed mode with a cycle time of 2 s. The number of microscans to be set at 1 scan s^−1^ (charge state 2–7) within an isolation window of 1.6 m/z were considered for MS/MS analysis. Dynamic exclusion was set at 30 s. Mass tolerance of ± 10 ppm was allowed, and the precursor intensity threshold was kept at 2.5E5.

### 2.11 Data analysis

All the MS raw data were processed by Maxquant 3.2.0 and searched against the homo sapiens in the UniProt database. The trypsin and Glu C cleavage with a maximum of two leakage sites was allowed. Oxidation on methionine (M), acetylation of protein N terminus, deamination (N) and phospho-modification (STY) were set as the variable modifications. Carbamidomethyl (C) was set as a fixed modification, The false discovery rate (FDR) was set at 1%. The other conditions were set by default.

## 3 Result and discussion

### 3.1 Synthesis and characterization of poly His modified silica microspheres

**FIGURE 1:**
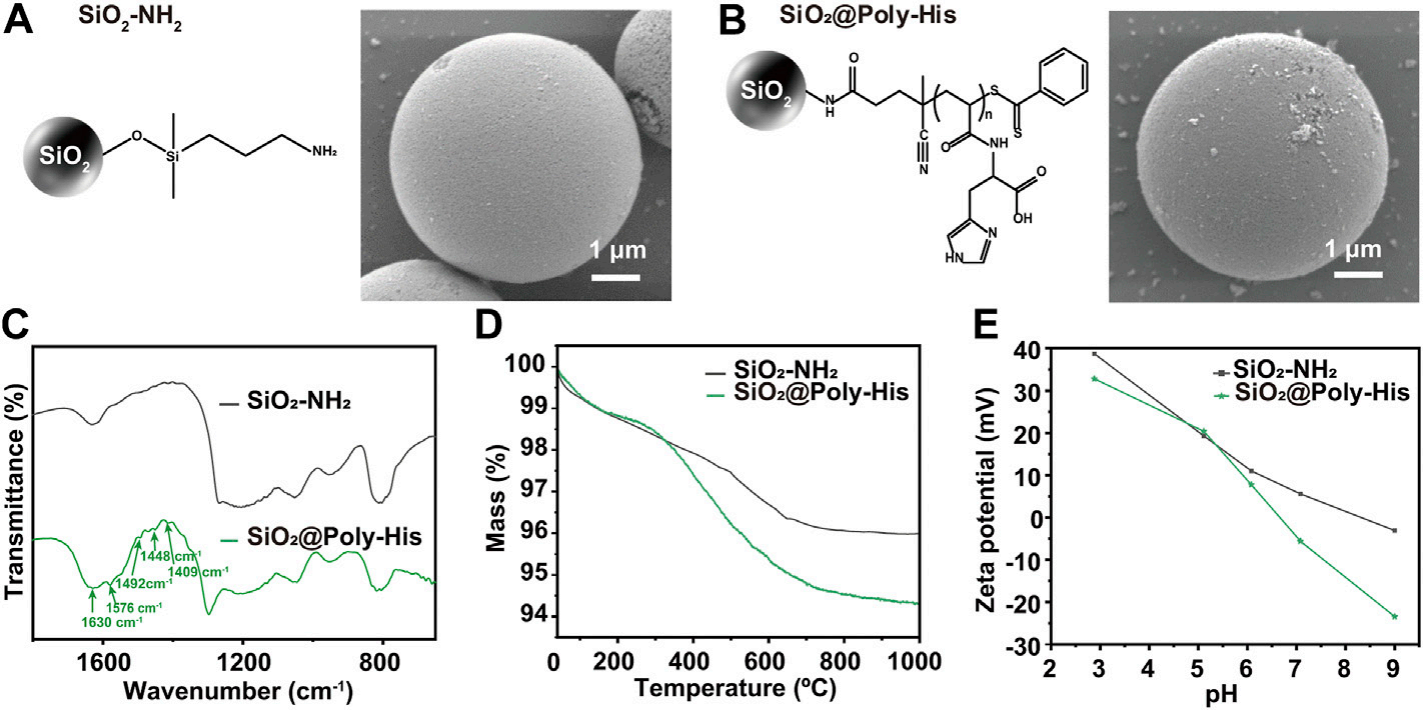
Characterization of SiO_2_@Poly-His microspheres. **(A)** SEM image of SiO_2_-NH_2_; **(B)** SEM image of SiO_2_@Poly-His microspheres; **(C)** FTIR spectra **(D)** TGA curves and **(E)** Zeta potential curves of SiO_2_-NH_2_ and SiO_2_@Poly-His microspheres.

We modified His monomer onto silica surface by surface initiated reversible addition-fragmentation transfer (SI-RAFT) polymerization (Raula et al., 2003) to obtained SiO_2_@Poly-His microspheres (schematically illustrated in Supplementary Figure S1A). After synthesis, the SiO_2_@Poly-His microspheres were characterized with different methods. Fourier transform interferometric radiometer (FTIR) spectrum (Figure 1C) shows that the strong adsorption at 1,630 cm^−1^ belongs to C = O group of carboxyl of His group. The peak at 1,576 cm^−1^ is attributed to the imidazole side ring stretching motions. The adsorptions around 1,409, 1,448, and 1,492 cm^−1^ are assigned to the C = C bond and the C-N bond of the imidazole ring. The morphology of SiO_2_@Poly-His microspheres characterized by SEM images show the morphology of microspheres has little change after modification (Figures 1A,B). According to thermogravimetric analysis, SiO_2_@Poly-His microspheres show 1.7% weight loss compared with SiO_2_-NH_2_ (Figure 1D) and exhibit a wider range of Zeta potential than amino silica. SiO_2_@Poly-His microspheres have lower Zeta potential at high pH (Figure 1E). The pore size distribution of the silica gel before (black) and after (bule) poly-His modification was investigated by N_2_ adsorption–desorption measurement. Barret-Joyner-Halenda (BJH) model was used for pore size distribution assessment. The result indicates that the film thickness of histidine polymer on SiO_2_ is −6 nm (Supplementary Figure S2). These results indicate that the SiO_2_@Poly-His microspheres were successfully prepared.

Subsequently, the SiO_2_@Poly-His microspheres with different initial feed molar ratio of His monomer to chain transfer reagent (CTA) were synthesized, their characterizations are shown in Supplementary Figure S1. Then we investigated the effect of the initial feed molar ratio of His monomer to CTA on the retention behavior and adsorption capacity of glycopeptides. When the initial feed molar ratio of His to CTA for SiO_2_@Poly-His microspheres increases from 5:1, 10:1 to 20:1, the retention behavior (Supplementary Figure S3) and adsorption capacity (Supplementary Figure S4) of glycopeptides increases accordingly. Therefore, the SiO_2_@Poly-His microspheres synthesized with an initial molar ratio of His to CTA of 20:1 were chosen for subsequent study.

### 3.2 Enrichment of glycopeptides with SiO_2_@Poly-His microspheres

**FIGURE 2:**
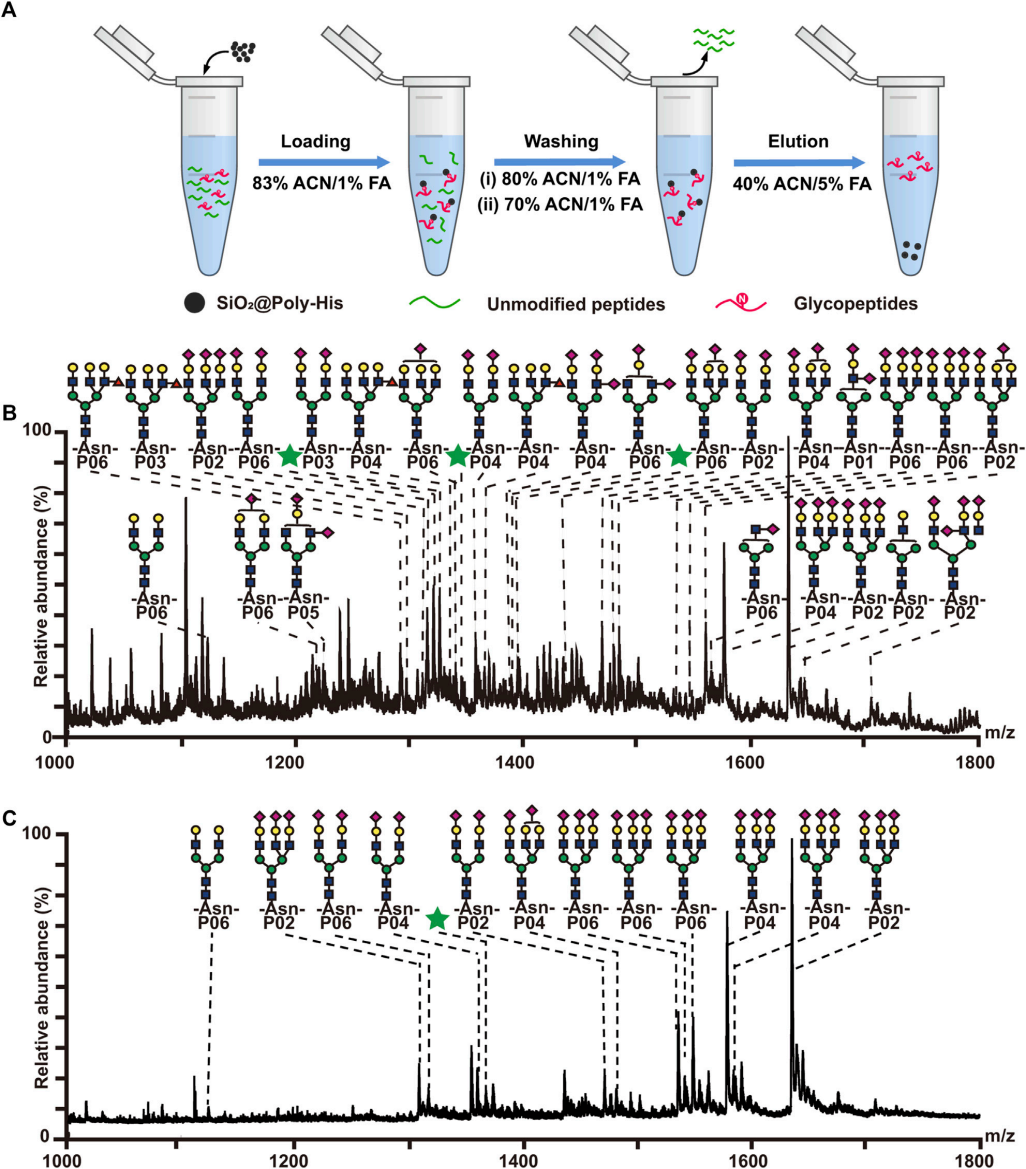
Enrichment of glycopeptides with SiO_2_@Poly-His microspheres and commercial ZIC-HILIC materials. **(A)** Workflow for the glycopeptide enrichment with the SiO_2_@Poly-His microspheres; **(B)** Mass spectrum of the mixture of the bovine fetuin and BSA at molar ratio of 1:5,000 enriched by SiO_2_@Poly-His microspheres; **(C)** Mass spectrum of the mixture of the bovine fetuin and BSA at molar ratio of 1:5,000 enriched by ZIC-HILIC. Glycopeptides are labeled with their glycan structures or green stars (glycopeptides with unknown glycoform): 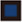: GlcNAc; 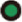: mannose; 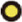: galactose; 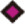: Neu5Ac.

Furthermore, the performance of SiO_2_@Poly-His microspheres for glycopeptides enrichment was tested using tryptic digests of bovine fetuin and bovine serum albumin (BSA) with different molar ratios. The procedure is shown in Figure 2A. After enrichment with SiO_2_@Poly-His microspheres, 30 glycopeptides (detailed information of glycopeptides in Supplementary Table S1, Supplementary Figure S5) are detected with high signal intensity from tryptic digests of bovine fetuin and BSA with the molar ratio of 1:100, in sharp contrast to none detected glycopeptide signal before SiO_2_@Poly-His treatment (Supplementary Figure S5). Even the molar ratio of bovine fetuin/BSA is dramatically decrease to 1:5,000, 29 glycopeptides could still be found dominating the spectrum (Figure 2B). As a comparison, the commercial ZIC-HILIC materials could only enrich 13 glycopeptides from the digests of bovine fetuin and BSA with the molar ratio of 1:200 (Figure 2C). Above results fully demonstrate that SiO_2_@Poly-His microspheres have higher selectivity toward glycopeptides than ZIC-HILIC

### 3.3 Enrichment of phosphopeptides with SiO_2_@Poly-His microspheres

**FIGURE 3:**
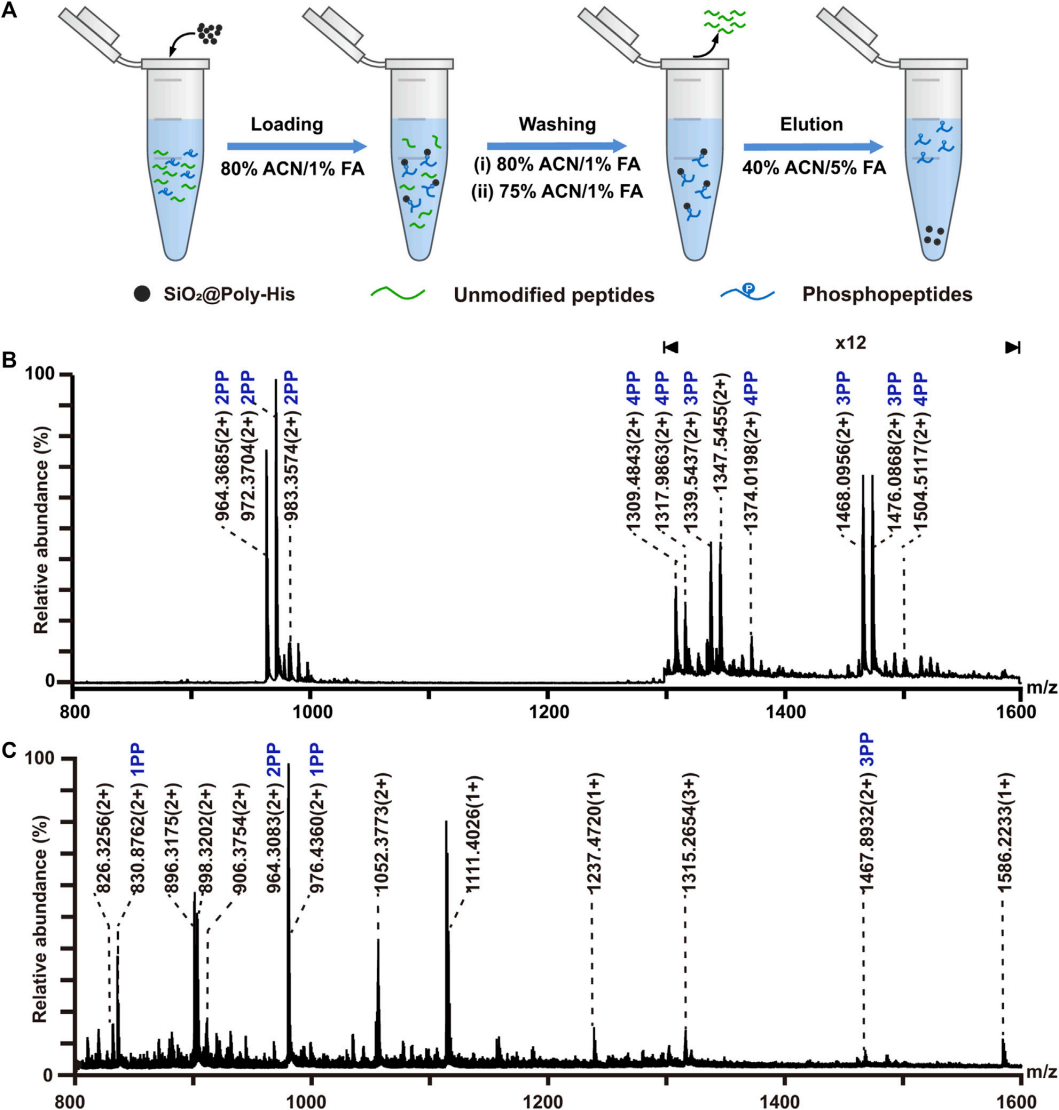
Enrichment of phosphopeptides with SiO_2_@Poly-His microspheres and commercial TiO_2_ materials. **(A)** Workflow for the phosphopeptides enrichment with SiO_2_@Poly-His microspheres; **(B)** Mass spectrum of the mixture of the casein and BSA at molar ratio of 1:1,000 enriched by SiO_2_@Poly-His microspheres; **(C)** Mass spectrum of the mixture of the casein and BSA at molar ratio of 1:1,000 enriched by TiO_2_. XPP represents phosphopeptide, among which X is the number of phosphate groups in a peptide; Only mass charge ratio (m/z) represents non-phosphopeptide.

According to literature reports (Chou et al., 2014; Chattopadhyay et al., 2016), His can interact with phosphate groups *via* multi-hydrogen bonding interactions. To investigate the interaction between SiO_2_@Poly-His microspheres and phosphopeptides, we investigated the retention of phosphopeptides on SiO_2_@Poly-His microspheres under step-wise ACN and FA content conditions, respectively (Supplementary Figure S6 and Supplementary Figure S7). The results indicate that hydrophilic interactions and hydrogen bonding interactions contribute to the retention of phosphopeptides on SiO_2_@Poly-His microspheres. Next, we investigated the performance of SiO_2_@Poly-His microspheres on phosphopeptides enrichment. The enrichment procedure is shown in Figure 3A. Eleven phosphopeptides (1 monophosphopeptide, 4 diphosphopeptides and 6 multi-phosphopeptides) dominating the spectrum (detailed information of phosphopeptides in Supplemntary Table S2) could be enriched from digests of α-casein (phosphoprotein) and BSA with the molar ratio of 1:100, in sharp contrast to none phosphopeptide before enrichment (Supplementary Figure S8). With decreasing the molar ratio of α-casein and BSA to 1:1,000, 10 phosphopeptides (3 diphosphopeptides and 7 multi-phosphopeptides) could still be observed (Figure 3B). So far, there was no report on the phosphopeptides enrichment by using any kind of amino acid modified materials, which could resist such high fold interference. As a comparison, the commercial TiO_2_ materials could enrich 4 phosphopeptides (2 monophosphopeptides, 1 diphosphopeptides and only 1 multi-phosphopeptide) from the same sample (Figure 3C). Besides, the number of multi-phosphopeptides enriched by SiO_2_@Poly-His microspheres is 7 times that of TiO_2_, demonstrating the preference of SiO_2_@Poly-His in enriching multi-phosphopeptides. In addition, the recovery toward phosphopeptides and sialylated glycopeptide were evaluated by SiO_2_@Poly-His microspheres based method, showing the preference of SiO_2_@Poly-His for di- and multi-phosphopeptides (Supplementary Figure S9). These results may be explained that the hydrophilic interactions and hydrogen bonding interactions between SiO_2_@Poly-His microspheres and phosphopeptides are more tunable than the Lewis acid-base interactions between TiO_2_ and phosphopeptides. These results demonstrate that SiO_2_@Poly-His microspheres have high selectivity and coverage toward phosphopeptides and are complementary to the TiO_2_, suggesting that these two methods can be used in combination in future.

### 3.4 Simultaneous enrichment of glycopeptides and phosphopeptides

**FIGURE 4:**
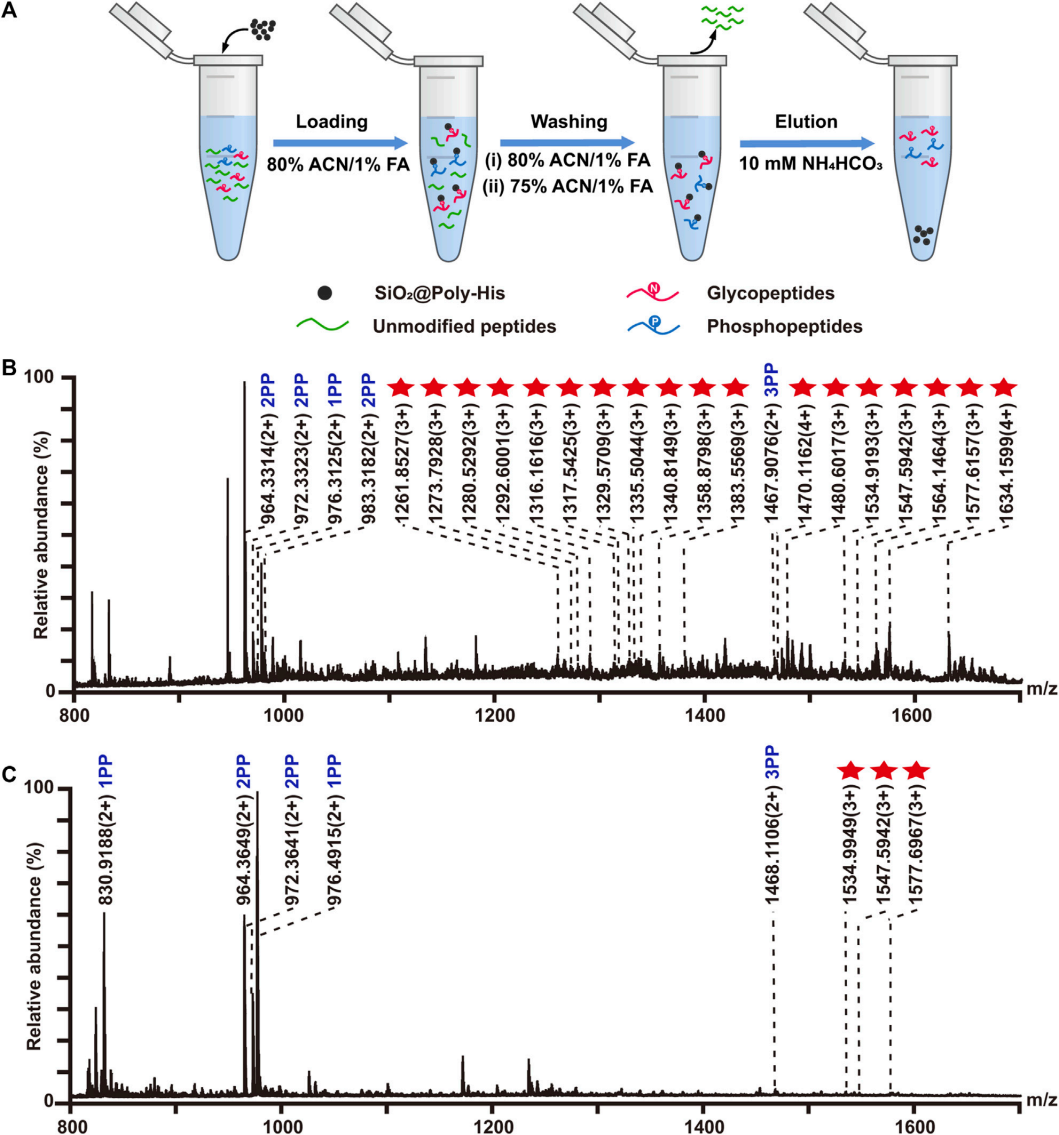
Simultaneous enrichment of glycopeptides and phosphopeptides with SiO_2_@Poly-His microspheres and commercial TiO_2_ materials. **(A)** Workflow for the simultaneous enrichment of glycopeptides and phosphopeptides with SiO_2_@Poly-His microspheres; **(B)** Mass spectrum of the mixture of α-casein, bovine fetuin and BSA at molar ratio of 1:0.67:1,000 enriched by SiO_2_@Poly-His microspheres and **(C)** Mass spectrum of the mixture of α-casein, bovine fetuin and BSA at molar ratio of 1:1:1,000 enriched by TiO_2_. Red star represents glycopeptide; XPP represents phosphopeptide, among which X is the number of phosphate groups in a peptide.

On the basis of above results, we attempted to employ SiO_2_@Poly-His microspheres to simultaneously enrich phosphopeptides and glycopeptides. The enrichment procedure is shown in Figure 4A. The protein digests of α-casein, bovine fetuin and BSA with the molar ratio of 1:0.67:1,000 are applied to simulate complex sample. The enrichment result shows that 20 glycopeptides and 5 phosphopeptides could be simultaneously detected (Figure 4B). It achieves simultaneous enrichment of phosphopeptides and glycopeptides at the highest fold of BSA interference reported to date (Supplementary Table S7), implying the outstanding selectivity of SiO_2_@Poly-His microspheres toward phosphopeptides and glycopeptides. When the sample of alpha-casein and BSA with the molar ratio of 1:1:1,000 is treated with TiO_2_ only 3 glycopeptides and 5 phosphopeptides are identified (Figure 4C). These results demonstrate that SiO_2_@Poly-His microspheres possess excellent performance for simultaneous enrichment of phosphopeptides and glycopeptides.

### 3.5 Investigating the hydrolysis degree of phosphopeptides during the deglycosylation process

In the reported protocols, the simultaneously enriched phosphopeptides and glycopeptides are always co-eluted and the co-eluates are deglycosylated by PNGase F to analyze glycosylation sites. Deglycosylation usually takes 10–16 h in ammonium bicarbonate aqueous (pH−8.0) solution (Palmisano et al., 2012; Boersema et al., 2013). During this deglycosylation process, the coexisted phosphopeptides were assumed to be hydrolyzed (Li et al., 2014), but no experimental data were reported. To investigate hydrolysis degree of phosphopeptides under deglycosylation process, standard monophosphopeptide (1 PP), diphosphopeptide (2 PP) and triphosphopeptide (3 PP) with the same peptide sequence (HSPIAPSSPSPK) are selected as model samples. To relatively quantify the hydrolysis of phosphopeptides in alkaline conditions, Glu-Fibrinopeptide B (GFP) is used as an internal standard. As the enzymolysis time became longer, the content of phosphopeptides decreased linearly, and the degree of phosphorylation status was positively correlated with its hydrolysis rate. After 12 h of enzymolysis, the content of 3 pp, 2 pp, 1 pp are dramatically reduce by 40%, 30%, 20%, respectively (Supplementary Figure S10). This result demonstrates that the phosphopeptides could be hydrolyzed during deglycosylation and multi-phosphopeptides are more labile for hydrolysis. To reduce/avoid the hydrolysis of the phosphopeptides in free solution, there are three ways to do: separating glycopeptides and phosphopeptides before deglycosylation, shortening deglycosylation time of co-eluates, and confining the phosphopeptides onto materials instead of in free solution.

### 3.6 On-line de-glycosylation and sequential elution of deglycosylated peptides and phosphopeptides

**FIGURE 5:**
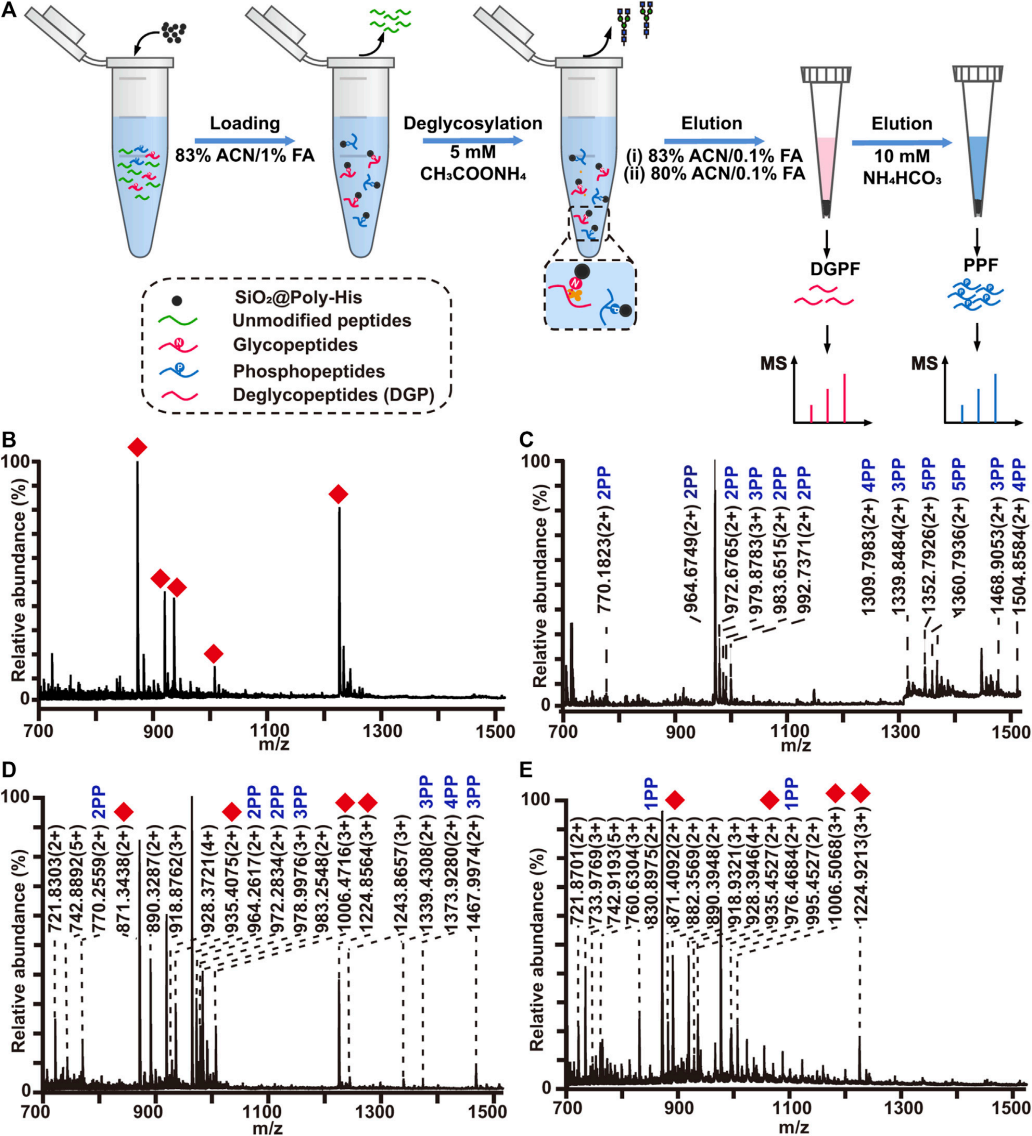
**(A)** Workflow the simultaneous enrichment with SiO_2_@Poly-His microspheres, which consists of the sequential elution of glycopeptides and phosphopeptides. Mass spectrum of sequential elution of the deglycosylated peptides fraction **(B)** and the phosphopeptides fraction **(C)** enriched by SiO_2_@Poly-His microspheres. Mass spectra of the co-elution of deglycosylated peptides and phosphopeptides that treated with SiO_2_@Poly-His microspheres **(D)** and commercial TiO_2_ materials **(E)**. Red diamond represents deglycosylated peptide; XPP represents phosphopeptide, among which X is the number of phosphate groups in a peptide; Only mass charge ratio (m/z) represents unmodified peptide.

To reduce the phosphopeptides hydrolysis during the process of deglycosylation treatment, we developed a strategy based on on-line deglycosylation and sequential elution of glycopeptides and phosphopeptides. Firstly, glycopeptides and phosphopeptides are co-enriched with SiO_2_@Poly-His microspheres; Then, the captured glycopeptides on the SiO_2_@Poly-His microspheres are deglycosylated with PNGase F and deglycopeptides are separated while phosphopeptides are still bound on the adsorbents. Finally, the bound phosphopeptides on SiO_2_@Poly-His microspheres are subsequently eluted (Figure 5A). To test this method, the digests of bovine fetuin and α-casein are used as samples. After treatment by as-described method, 5 deglycopeptides and 12 phosphopeptides (5 diphosphopeptides and 7 multi-phosphopeptides) are detected, respectively (Figures 5B,C). As comparison, 4 deglycopeptides and 7 phosphopeptides (3 diphosphopeptides and 4 multi-phosphopeptides) are detected by using SiO_2_@Poly-His microspheres with co-elution strategy (Figure 5D). Obviously, the former one identifies more target PTM peptides, which means that more information could be obtained by our established strategy. Meanwhile, the commercial materials of TiO_2_ are also chosen as a comparison. Only 4 deglycopeptides and 2 monophosphopeptides are detected by using co-elution strategy (Figure 5E). Therefore, this strategy could not only achieve the sequential elution of glycopeptides and phosphopeptides, but also detect more target peptides, especially multi-phosphopeptides.

### 3.7 Analysis of phosphopeptides and glycopeptides from HT29 cell lysate

**FIGURE 6:**
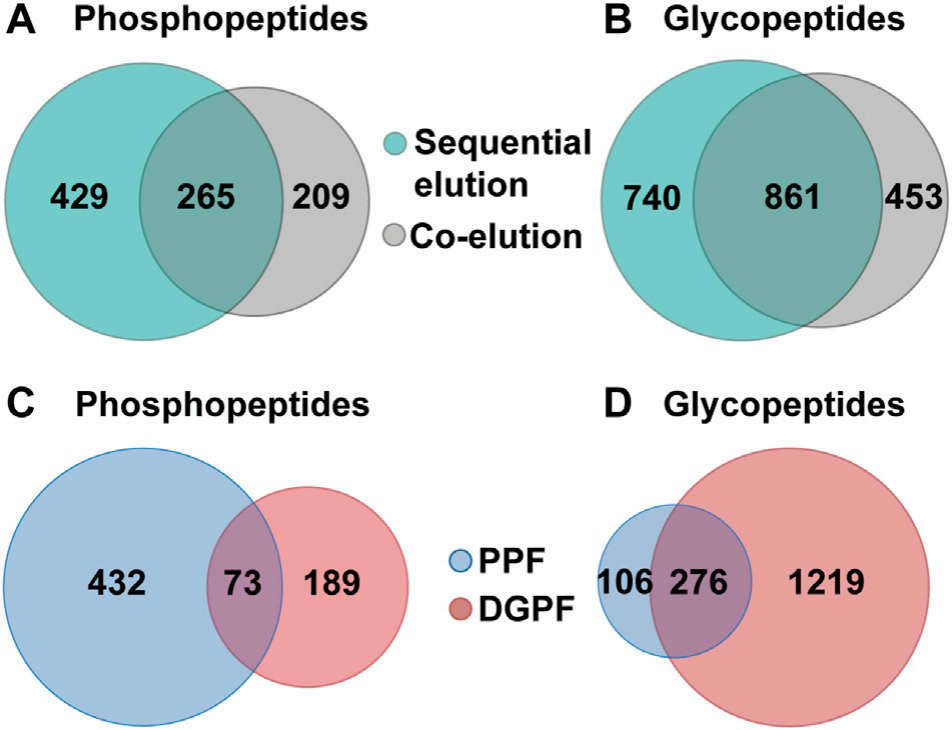
Simultaneous enrichment and sequential elution or co-elution of glycopeptides and phosphopeptides from HT29 cell lysates with SiO_2_@Poly-His microspheres. **(A)** The overlap of phosphopeptides between the sequential elution strategy and co-elution strategy; **(B)** The overlap of glycopeptides between the sequential elution strategy and co-elution strategy; **(C)** The overlap of phosphopeptides between deglycosylated peptide fraction and phosphopeptide fraction; **(D)** the overlap of glycopeptides between deglycosylated peptide fraction and phosphopeptide fraction.

We further applied the SiO_2_@Poly-His microspheres based on-line deglycosylation strategy to simultaneously enrich and sequentially separate phosphopeptides and glycopeptides from HT-29 cell lysates. Deglycosylated peptide fraction and phosphopeptide fraction were obtained. For comparison, the same samples are treated with SiO_2_@Poly-His microspheres and the target peptides are co-eluted and further deglycosylated (co-elution strategy). By using our established strategy, 1,601 glycopeptides (Figure 6A, Supplementary Table S3) and 694 phosphopeptides (Figure 6B, Supplementary Table S4) are identified from 100 μg HT29 cell lysate. Among the identified phosphopeptides, the proportions of monophosphopeptides, diphosphopeptides and multi-phosphopeptides are 53.5%, 35.7%, and 10.8%, respectively (Supplementary Figure S11). The numbers of the phosphopeptides in deglycosylated peptide and phosphopeptide fractions are 262 and 505, respectively (Figure 6C). The overlap of phosphopeptides between these fractions is 10%. In deglycosylated peptide fraction, the proportions of monophosphopeptides, diphosphopeptides and multi-phosphopeptides are 78.4%, 20.5%, and 1.1%, respectively. While in phosphopeptide fraction, the proportions of monophosphopeptides, diphosphopeptides and multi-phosphopeptides are 41.5%, 43.1%, and 15.4%, respectively. Meanwhile, the numbers of the glycopeptides in deglycosylated peptide fraction and phosphopeptide fraction are 1,555 and 382, respectively (Figure 6D). The overlap of glycopeptides between deglycosylated peptide fraction and phosphopeptide fraction is 17%. These results suggest a low degree of overlap between deglycosylated peptide fraction and phosphopeptide fraction in sequential elution strategy. In contrast to these, only 1,314 glycopeptides (Supplementary Table S5) and 474 phosphopeptides (Supplementary Table S6) are identified from the identical sample using the co-elution strategy, among which the proportions of monophosphopeptides, diphosphopeptides and multi-phosphopeptides are 56.7%, 34.6%, and 8.7%, respectively (Supplementary Figure S12). The total number of phosphopeptides and glycopeptides identified by the sequential elution strategy is 1.5-fold and 1.2-fold higher than that of the co-elution strategy, respectively. These results demonstrate that our established strategy could simultaneously capture as well as sequentially separate glycopeptides and phosphopeptides, and increase the identified number of target peptides.

## 4 Conclusion

In summary, we developed one strategy to simultaneously enrich, on-line deglycosylate and sequentially elute phosphopeptides and glycopeptides based on SiO_2_@Poly-His microspheres. First, SiO_2_@Poly-His microspheres exhibit excellent selectivity for glycopeptides and phosphopeptide, providing a prerequisite for high coverage in global PTMs-proteomics. Second, the tunable interaction between the SiO_2_@Poly-His microspheres and PTM peptides will provide versatile candidates for the highly effective separation of various specific PTM peptides, especially for multi-phosphopeptides. Third, the on-line deglycosylation strategy reduces the hydrolysis loss of phosphopeptides and the suppression of low-abundance PTM peptides during MS analysis. We believe that the biomimetic SiO_2_@Poly-His microspheres may shed light on widespread applications ranging from biomolecule adsorption, biomarker and drug target discovery, and other biomedical fields.

## Data Availability

The raw data supporting the conclusions of this article will be made available by the authors, without undue reservation, to any qualified researcher.
