# Supplementary material for: Simultaneous enrichment and sequential separation of glycopeptides and phosphopeptides with poly-histidine functionalized microspheres
Source: Front Bioeng Biotechnol. 2022 Oct 6;10:1011851. doi: 10.3389/fbioe.2022.1011851 (PMC9582455; doi:10.3389/fbioe.2022.1011851)
Supplement: Supplementary file 7 [file DataSheet1.docx]

Supplementary Material

Table of Contents

Figure S1 2

Figure S2 3

Figure S3 4

Figure S4 5

Figure S5 6

Figure S6 7

Figure S7 8

Figure S8 9

Figure S9 10

Figure S10 11

Figure S11 12

Figure S12 13

Table S7 | An overview on recent reported the selectivity of materials for simultaneous enrichment

of glycopeptides and phosphopeptides. 14


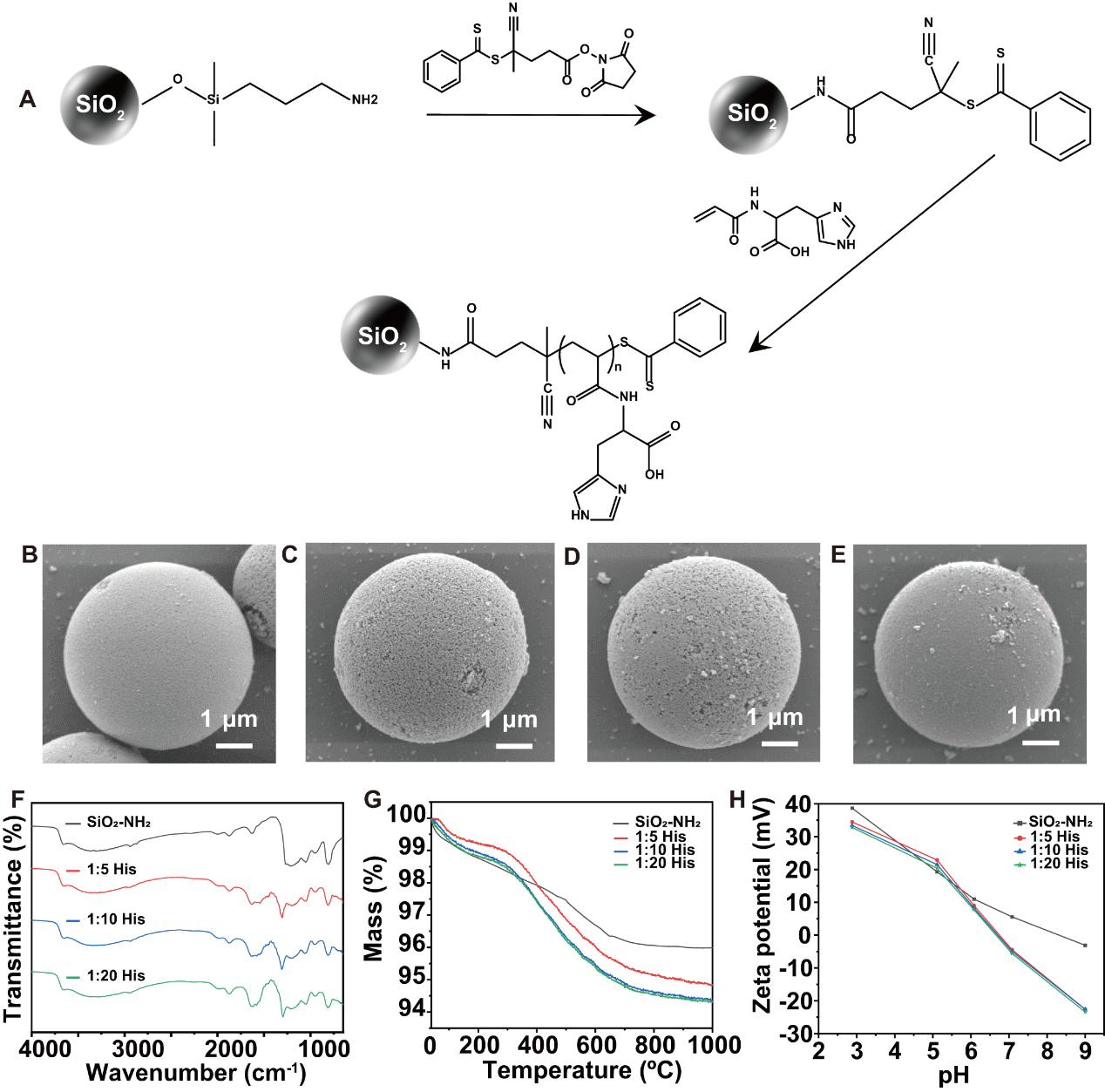


**Figure S1** | Synthesis process and characterization of SiO_2_@Poly-His microspheres. (A) Scheme of synthesis process; SEM characterization of SiO_2_-NH_2_ microspheres (A) and SiO_2_@Poly-His microspheres with different feed molar ratio of monomer histidine (His) varied from 5:1 (C), 10:1 (D) to 20:1 (E); IR spectrum (F), TGA curve (G) and Zeta potential curve (H) of SiO_2_@Poly-His microspheres.


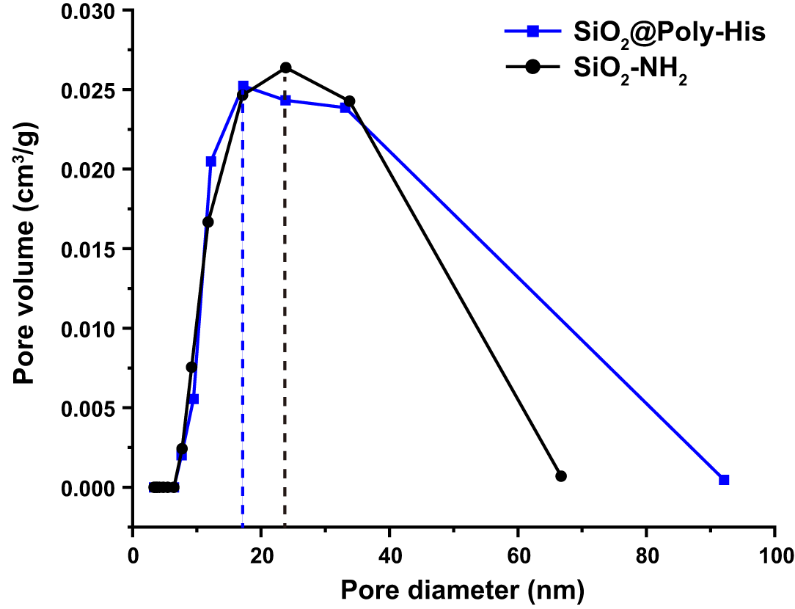


Figure S2 | Pore size distribution curve of the SiO_2_@Poly-His and SiO_2_-NH_2_ microspheres.

_
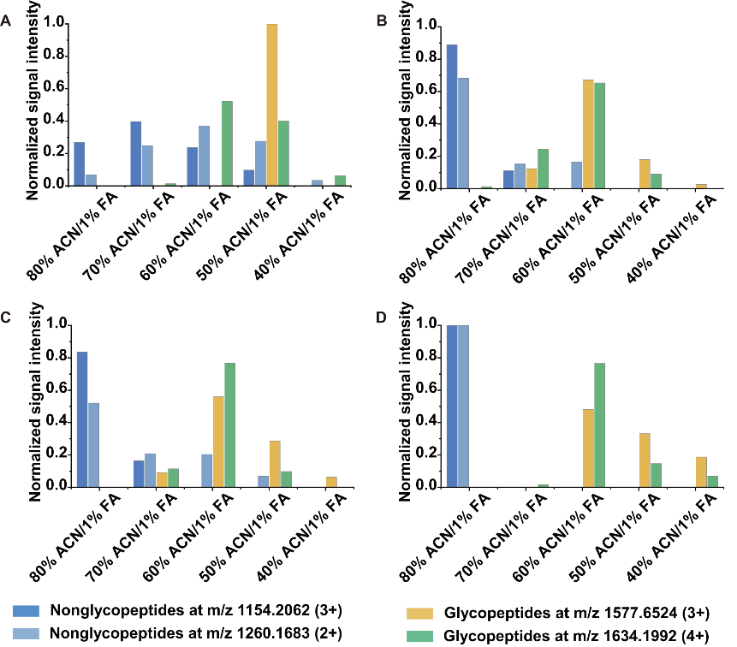
_

**Figure S3** | The retention behavior of peptides on SiO_2_-NH_2_ microspheres (A) and SiO_2_@Poly-His microspheres with different feed molar ratio of monomer His (B-D). 5 His:1 (B), 10 His:1 (C) and 20 His:1 (D).

**Figure S4** | The adsorption capacity of glycopeptides from bovine fetuin digests on SiO_2_@Poly-His microspheres with different feed molar ratio of monomer His.


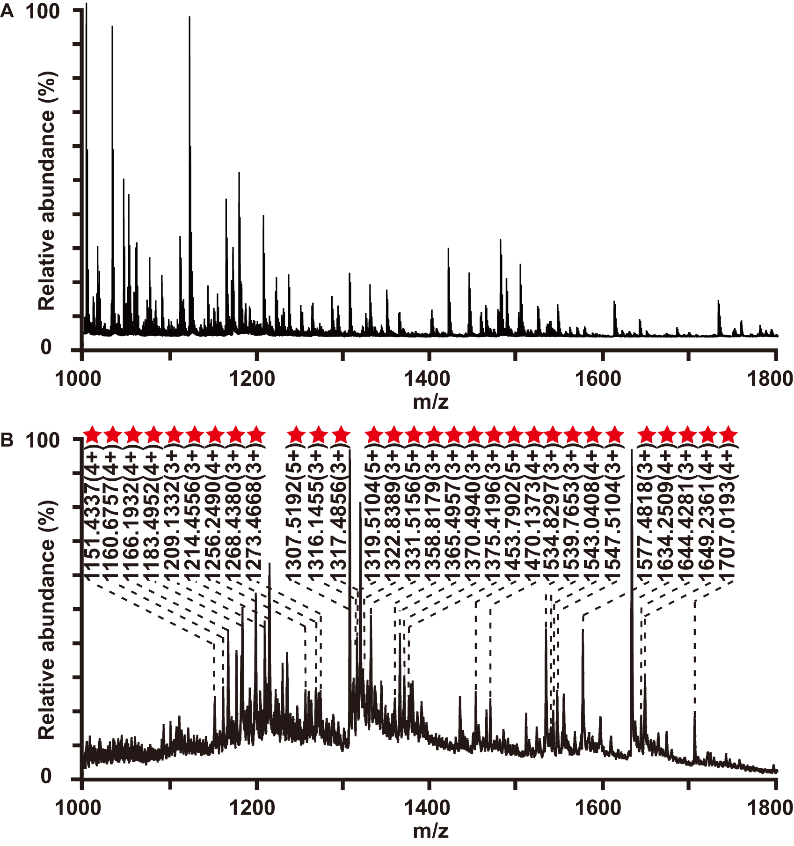


**Figure S5** | Mass spectra of digests of the bovine fetuin and BSA at molar ratio of 1:100 before (A) and after (B) enrichment with SiO_2_@Poly-His microspheres. Red star represents glycopeptide.


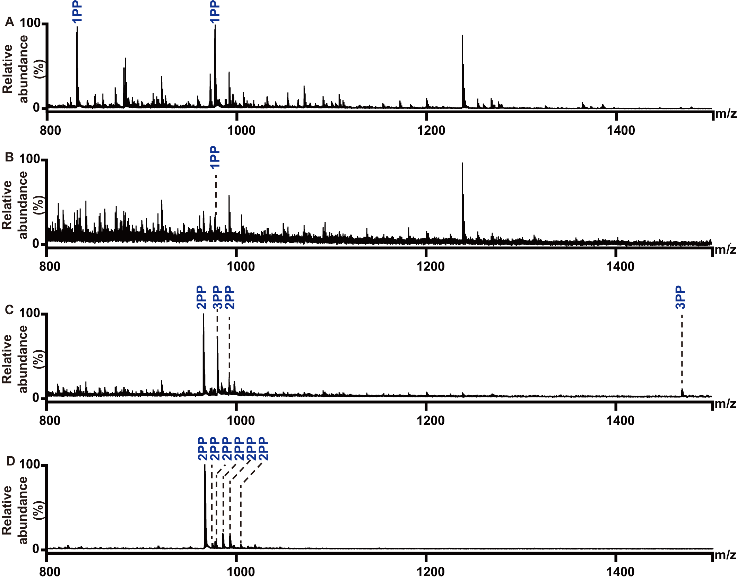


**Figure S6** | The retention of phosphopeptides on SiO_2_@Poly-His microspheres with different content of ACN. (A) 80% ACN/1% FA; (B) 70% ACN/1% FA; (C) 60% ACN/1% FA; (D) 50% ACN/1% FA. XPP represents phosphopeptide, among which X is the number of phosphate groups in a peptide.


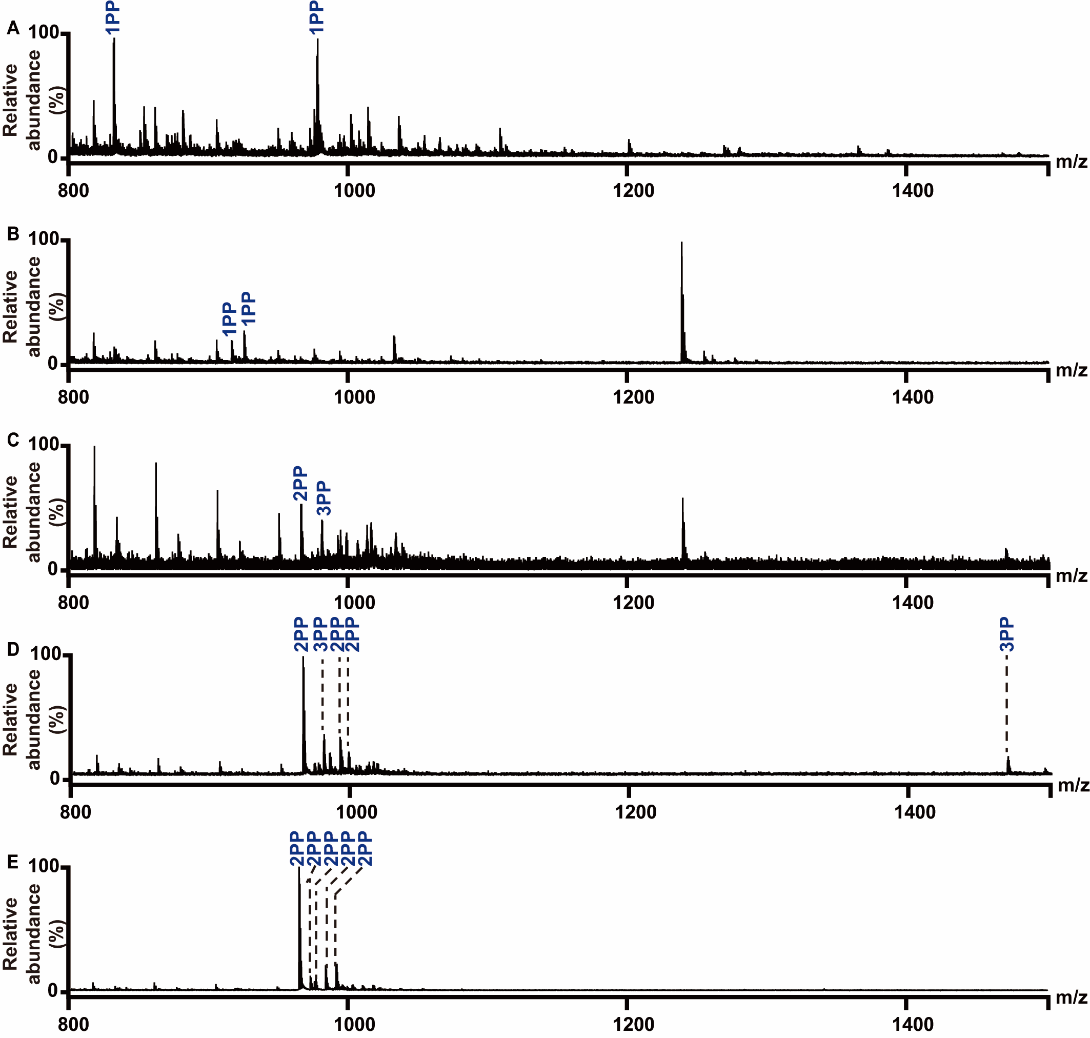


**Figure S7** | The retention of phosphopeptides on SiO_2_@Poly-His microspheres with different content of FA. (A) 70% ACN/0.1% FA; (B) 70% ACN/0.5% FA; (C) 70% ACN/1% FA; (D) 70% ACN/2% FA, (E) 70% ACN/5% FA. XPP represents phosphopeptide, among which X is the number of phosphate groups in a peptide.


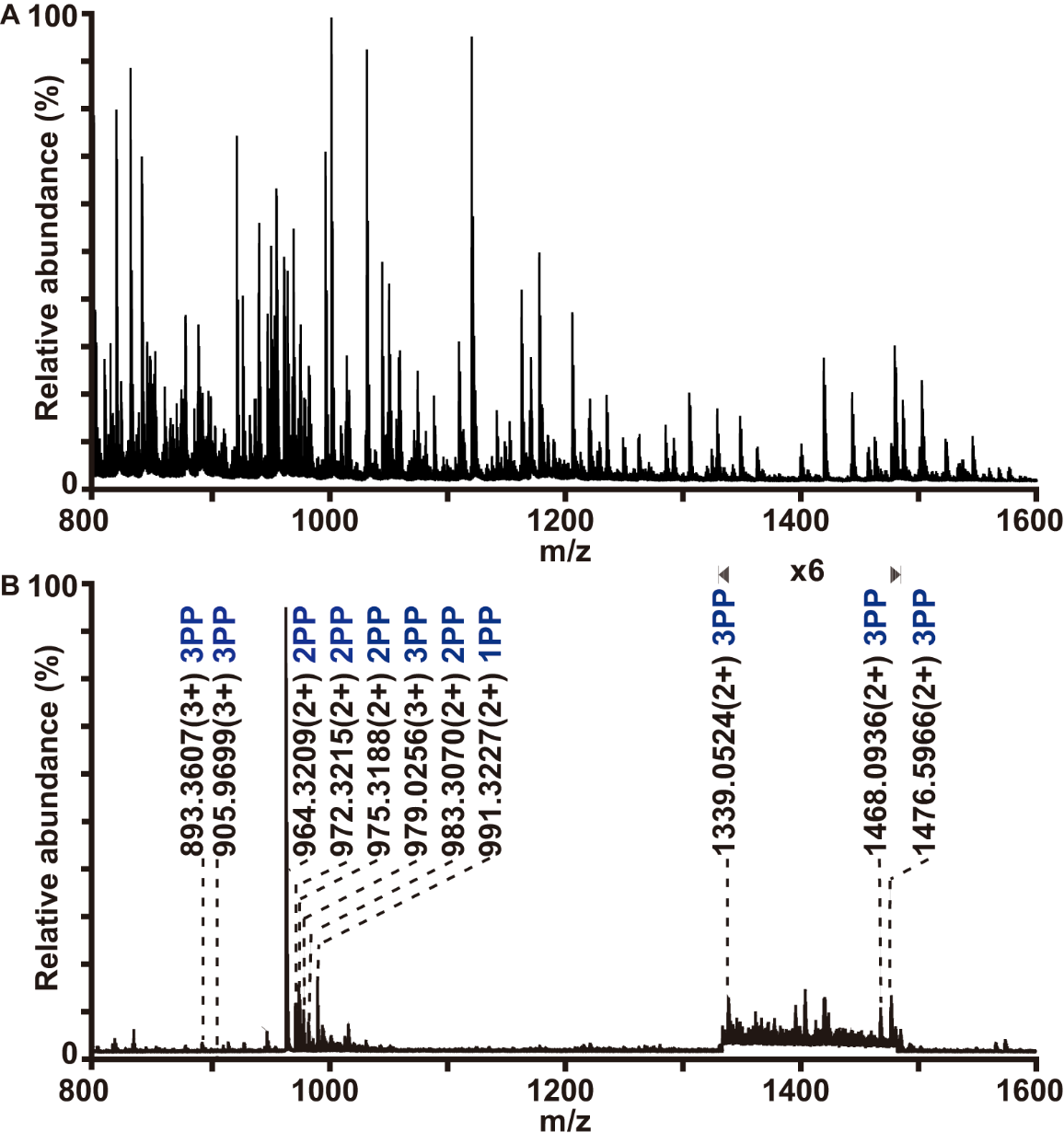


**Figure S8** | Mass spectra of the digests of the α-casein and BSA at molar ratio of 1:100 before (A) and after (B) enrichment with SiO_2_@Poly-His microspheres. XPP represents phosphopeptide, among which X is the number of phosphate groups in a peptide.

**Figure S9** | The recovery of phosphopeptides and sialylated glycopeptide with SiO_2_@Poly-His microspheres based method. The recovery was determined by stable-isotope dimethyl labeling (Sun et al., 2012).


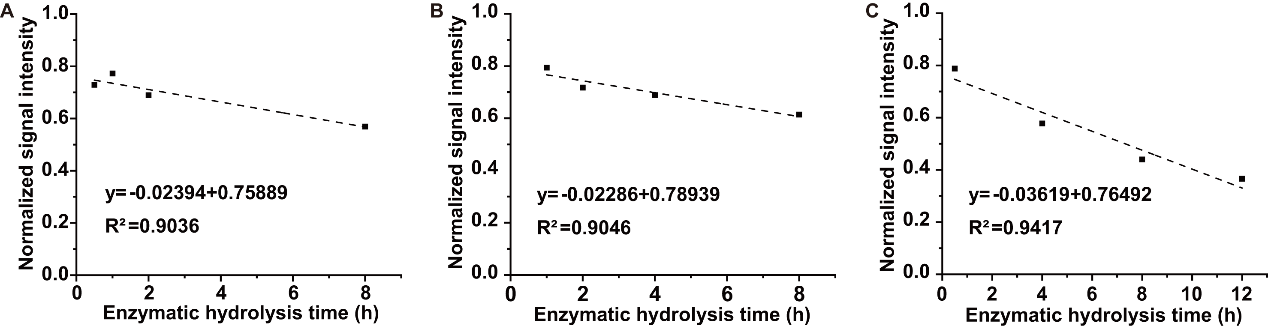


**Figure S10** | Hydrolysis degree of phosphopeptide standards over time during the deglycosylation process. (A) Monophosphopeptide (HS^*^PIAPSSPSPK); (B) diphosphorylated peptide (HS^*^PIAPSSP S^*^PK); (c) triphosphopeptide (HS^*^PIAPS S^*^P S^*^PK).


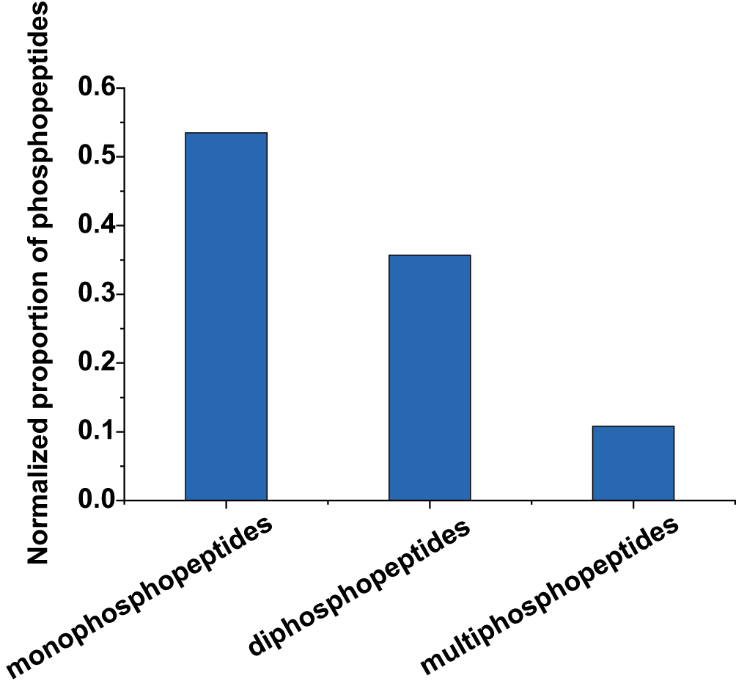


**Figure S11** | Proportion of phosphopeptides with different phosphorylation degree enriched from HT29 cell lysates with SiO_2_@Poly-His microspheres based sequential elution strategy.


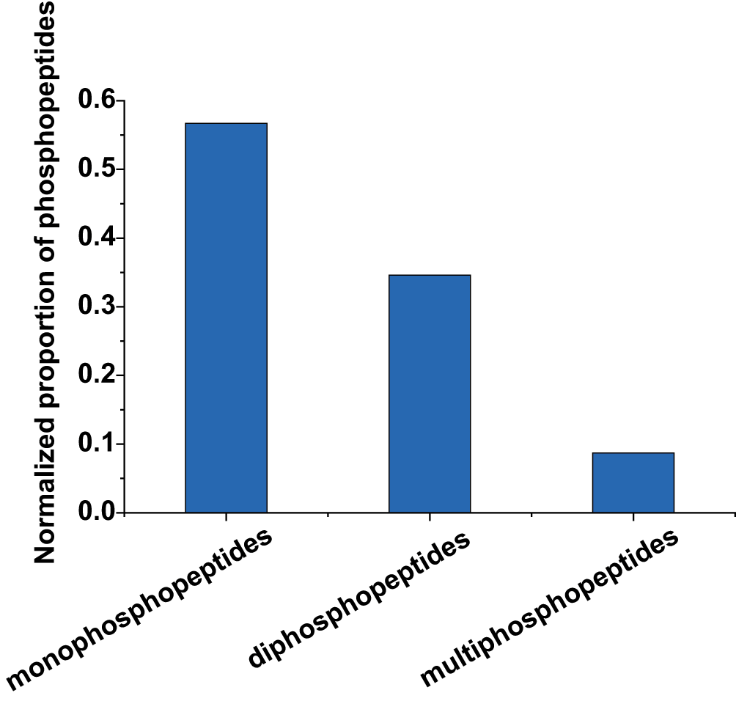
­

**Figure S12** | Proportion of phosphopeptides with different phosphorylation degree enriched from HT29 cell lysates with SiO_2_@Poly-His microspheres based co-elution strategy.

**Table S7 |** An overview on recent reported the selectivity of materials for simultaneous enrichment of glycopeptides and phosphopeptides.

| Materials | Selectivity | | | Ref. |
| --- | --- | --- | --- | --- |
|  | PP | GP | PP+GP |  |
| co-PAN@Ti^4+^ | - | - | 1:1:50 | (Wang et al., 2016) |
| TiO_2_@SiO_2_-B(OH)_2_@Fe_3_O_4_@TiO_2_ | 1:1000 | 1:50 | - | (Xu et al., 2016) |
| SiO_2_–NH_2_@TiO_2_ | - | - | 1:2:200 | (Lin et al., 2017) |
| Fe_3_O_4_@PDA@ UiO-66-NH_2_ | 1:500 | 1:500 | - | (Xie and Deng, 2017) |
| SiO_2_–NH_2_@TiO_2_ | - | - | 1:1:500 | (Xu et al., 2017) |
| CS@PGMA@IDA-Ti^4+^ | 1:5000 | 1:100 | - | (Zou et al., 2017) |
| MagG@PEI@PA-Ti^4+^ | 1:5000 | 1:1000 | - | (Hong et al., 2018) |
| Fe_3_O_4_@MIL-100(Fe) | 1:500 | 1:500 | - | (Wu et al., 2018) |
| Mag-MSMs@PEI-PA-Ti^4+^ | 1:5000 | 1:1000 | - | (Hong et al., 2019) |
| magG@PDA@UiO-66-NH_2_. | 1:1000 | 1:200 | - | (Liu et al., 2019) |
| SPIOs@SiO_2_@MOF | 1:400 | 1:500 | - | (Luo et al., 2019) |
| Fe_3_O_4_@mTiO_2_-MSA | 1:800 | 1:100 | - | (Sun et al., 2019a) |
| Mag TiO_2_-GSH | 1:1000 | 1:100 | - | (Sun et al., 2019b) |
| mMIL-125@Au@L-Cys | 1:100 | 1:100 |  | (Wu et al., 2019) |
| PNI-co-ATBA_0.2_@SiO_2_ | - | 1:500 | 1:3:193 | (Lu et al., 2020) |
| Fe_3_O_4_@TiO_2_-IDA | 1:800 | 1:100 | - | (Sun et al., 2020) |
| Fe^3+^ immobilized polySD-SiO_2_ | - | - | 1:2:400 | (Zhang et al., 2020) |
| magOTfP5SOF-Ga^3+^ | 1:1000 | 1:2000 | 1:1:1 | (Zheng et al., 2020) |
| Fe_3_O_4_@PDA@mTiO_2_@PEI-g-ZIF-8 | 1:1000 | 1:1000 | - | (Yi et al., 2020) |
| MCNC@Polymer@COF-MUBA | - | - | 1:1:1000 | (Luo et al., 2021) |
| FZT-GSH | 1:500 | 1:50 | - | (Chu et al., 2022) |
| HHZr-MOFs | 1:2000 | 1:1000 | - | (He et al., 2022) |
| GO@CS@ZIF‑8 foam | - | - | 1:1:500 | (Liu et al., 2022) |
| SiO_2_@Poly-His | 1:1000 | 1:5000 | 1:0.67:1000 | This work |

**REFERENCES**

Chu, H., Zheng, H., Sun, N., and Deng, C. (2022). Simultaneous analysis of cellular glycoproteome and phosphoproteome in cervical carcinoma by one-pot specific enrichment. *Anal. Chim. Acta* 1195. doi: 10.1016/j.aca.2021.338693.

He, Y., Zheng, Q., Huang, H., Ji, Y., and Lin, Z. (2022). Synergistic synthesis of hydrophilic hollow zirconium organic frameworks for simultaneous recognition and capture of phosphorylated and glycosylated peptides. *Anal. Chim. Acta* 1198. doi: 10.1016/j.aca.2022.339552.

Hong, Y., Zhan, Q., Zheng, Y., Pu, C., Zhao, H., and Lan, M. (2019). Hydrophilic phytic acid-functionalized magnetic dendritic mesoporous silica nanospheres with immobilized Ti^4+^: A dual-purpose affinity material for highly efficient enrichment of glycopeptides/phosphopeptides. *Talanta* 197**,** 77-85. doi: 10.1016/j.talanta.2019.01.005.

Hong, Y., Zhao, H., Pu, C., Zhan, Q., Sheng, Q., and Lan, M. (2018). Hydrophilic phytic acid-coated magnetic graphene for titanium(iv) immobilization as a novel hydrophilic interaction liquid chromatography-immobilized metal affinity chromatography platform for glyco- and phosphopeptide enrichment with controllable selectivity. *Anal. Chem.* 90(18)**,** 11008-11015. doi: 10.1021/acs.analchem.8b02614.

Lin, H., Yuan, K., and Deng, C. (2017). Preparation of a TiO_2_-NH_2_ modified MALDI plate for on-plate simultaneous enrichment of phosphopeptides and glycopeptides. *Talanta* 175**,** 427-434. doi: 10.1016/j.talanta.2017.07.078.

Liu, B., Lu, Y., Wang, B., Yan, Y., Liang, H., and Yang, H. (2019). Facile preparation of hydrophilic dual functional magnetic metal-organic frameworks as a platform for proteomics research. *Chemistryselect* 4(7)**,** 2200-2204. doi: 10.1002/slct.201803527.

Liu, R., Gao, W., Yang, J., Zhang, S., Wang, C., Lin, J., et al. (2022). A novel graphene oxide/chitosan foam incorporated with metal-organic framework stationary phase for simultaneous enrichment of glycopeptide and phosphopeptide with high efficiency. *Anal. Bioanal. Chem.* 414(6)**,** 2251-2263. doi: 10.1007/s00216-021-03861-z.

Lu, Q., Chen, C., Xiong, Y., Li, G., Zhang, X., Zhang, Y., et al. (2020). High-efficiency phosphopeptide and glycopeptide simultaneous enrichment by hydrogen bond-based bifunctional smart polymer. *Anal. Chem.* 92(9)**,** 6269-6277. doi: 10.1021/acs.analchem.9b02643.

Luo, B., Chen, Q., He, J., Li, Z., Yu, L., Lan, F., et al. (2019). Boronic acid-functionalized magnetic metal-organic frameworks via a dual-ligand strategy for highly efficient enrichment of phosphopeptides and glycopeptides. *ACS Sustainable Chem. Eng.* 7(6)**,** 6043-6052. doi: 10.1021/acssuschemeng.8b06171.

Luo, B., Yan, S., Zhang, Y., Zhou, J., Lan, F., and Wu, Y. (2021). Bifunctional magnetic covalent organic framework for simultaneous enrichment of phosphopeptides and glycopeptides. *Anal. Chim. Acta* 1177. doi: 10.1016/j.aca.2021.338761.

Sun, N., Wang, J., Yao, J., Chen, H., and Deng, C. (2019a). Magnetite nanoparticles coated with mercaptosuccinic acid-modified mesoporous titania as a hydrophilic sorbent for glycopeptides and phosphopeptides prior to their quantitation by LC-MS/MS. *Microchim. Acta* 186(3). doi: 10.1007/s00604-019-3274-3.

Sun, N., Wang, Z., Wang, J., Chen, H., Wu, H., Shen, S., et al. (2019b). Hydrophilic tripeptide combined with magnetic titania as a multipurpose platform for universal enrichment of phospho- and glycopeptides. *J. Chromatogr. A* 1595**,** 1-10. doi: 10.1016/j.chroma.2019.02.039.

Sun, N., Wu, H., and Shen, X. (2020). Magnetic titanium dioxide nanomaterial modified with hydrophilic dicarboxylic ligand for effective enrichment and separation of phosphopeptides and glycopeptides. *Microchim. Acta* 187(3). doi: 10.1007/s00604-020-4161-7.

Sun, Z., Qin, H., Wang, F., Cheng, K., Dong, M., Ye, M., et al. (2012). Capture and dimethyl labeling of glycopeptides on hydrazide beads for quantitative glycoproteomics analysis. *Anal. Chem.* 84(20)**,** 8452-8456. doi: 10.1021/ac302130r.

Wang, J., Wang, Y., Gao, M., Zhang, X., and Yang, P. (2016). Facile synthesis of hydrophilic polyamidoxime polymers as a novel solid-phase extraction matrix for sequential characterization of glyco- and phosphoproteomes. *Anal. Chim. Acta* 907**,** 69-76. doi: 10.1016/j.aca.2015.12.015.

Wu, Y., Liu, Q., and Deng, C. (2019). L-cysteine-modified metal-organic frameworks as multifunctional probes for efficient identification of N-linked glycopeptides and phosphopeptides in human crystalline lens. *Anal. Chim. Acta* 1061**,** 110-121. doi: 10.1016/j.aca.2019.01.052.

Wu, Y., Liu, Q., Xie, Y., and Deng, C. (2018). Core-shell structured magnetic metal-organic framework composites for highly selective enrichment of endogenous N-linked glycopeptides and phosphopeptides. *Talanta* 190**,** 298-312. doi: 10.1016/j.talanta.2018.08.010.

Xie, Y., and Deng, C. (2017). Designed synthesis of a "One for Two" hydrophilic magnetic amino-functionalized metal-organic framework for highly efficient enrichment of glycopeptides and phosphopeptides. *Sci. Rep.* 7. doi: 10.1038/s41598-017-01341-y.

Xu, D., Gao, M., Deng, C., and Zhang, X. (2016). Synthesis of bifunctional TiO2@SiO2-B(OH)2@Fe3O4@TiO2 sandwich-like nanosheets for sequential selective enrichment of phosphopeptides and glycopeptides for mass spectrometric analysis. *Anal. Bioanal Chem.* 408(20)**,** 5489-5497. doi: 10.1007/s00216-016-9647-0.

Xu, D., Yan, G., Gao, M., Deng, C., and Zhang, X. (2017). Highly selective SiO_2_-NH_2_@TiO_2_ hollow microspheres for simultaneous enrichment of phosphopeptides and glycopeptides. *Anal. Bioanal. Chem.* 409(6)**,** 1607-1614. doi: 10.1007/s00216-016-0101-0.

Yi, L., Yan, Y., Tang, K., and Ding, C.-F. (2020). Facile preparation of polymer-grafted ZIF-8-modified magnetic nanospheres for effective identification and capture of phosphorylated and glycosylated peptides. *Anal. Methods* 12(38)**,** 4657-4664. doi: 10.1039/d0ay01412e.

Zhang, Y., Li, J., Yu, Y., Xie, R., Liao, H., Zhang, B., et al. (2020). Coupling hydrophilic interaction chromatography materials with immobilized Fe(3+)for phosphopeptide and glycopeptide enrichment and separation. *RSC Adv.* 10(37)**,** 22176-22182. doi: 10.1039/d0ra01048k.

Zheng, H., Jia, J., Li, Z., and Jia, Q. (2020). Bifunctional magnetic supramolecular-organic framework: a nanoprobe for simultaneous enrichment of glycosylated and phosphorylated peptides. *Anal. Chem.* 92(3)**,** 2680-2689. doi: 10.1021/acs.analchem.9b04691.

Zou, X., Jie, J., and Yang, B. (2017). Single-step enrichment of n-glycopeptides and phosphopeptides with novel multifunctional Ti(4+)-immobilized dendritic polyglycerol coated chitosan nanomaterials. *Anal. Chem.* 89(14)**,** 7520-7526. doi: 10.1021/acs.analchem.7b01209.
